# Deciphering the Evolution and Development of the Cuticle by Studying Lipid Transfer Proteins in Mosses and Liverworts

**DOI:** 10.3390/plants7010006

**Published:** 2018-01-15

**Authors:** Tiina A. Salminen, D. Magnus Eklund, Valentin Joly, Kristina Blomqvist, Daniel P. Matton, Johan Edqvist

**Affiliations:** 1 Structural Bioinformatics Laboratory, Biochemistry, Faculty of Science and Engineering, Åbo Akademi University, FI-20520 Turku, Finland; tsalmine@abo.fi; 2 Department of Plant Ecology and Evolution, Evolutionary Biology Centre, Uppsala University, 752 36 Uppsala, Sweden; magnus.eklund@ebc.uu.se; 3 Institut de Recherche en Biologie Végétale, Département de Sciences biologiques, Université de Montréal, Montréal, QC H1X 2B2, Canada; valentin.joly@umontreal.ca (V.J.); dp.matton@umontreal.ca (D.P.M.); 4 IFM, Linköping University, 581 83 Linköping, Sweden; kristina.blomqvist@eskilstuna.se

**Keywords:** LTP, moss, lipids, liverwort, *Marchantia*, cutin, cuticle, wax

## Abstract

When plants conquered land, they developed specialized organs, tissues, and cells in order to survive in this new and harsh terrestrial environment. New cell polymers such as the hydrophobic lipid-based polyesters cutin, suberin, and sporopollenin were also developed for protection against water loss, radiation, and other potentially harmful abiotic factors. Cutin and waxes are the main components of the cuticle, which is the waterproof layer covering the epidermis of many aerial organs of land plants. Although the in vivo functions of the group of lipid binding proteins known as lipid transfer proteins (LTPs) are still rather unclear, there is accumulating evidence suggesting a role for LTPs in the transfer and deposition of monomers required for cuticle assembly. In this review, we first present an overview of the data connecting LTPs with cuticle synthesis. Furthermore, we propose liverworts and mosses as attractive model systems for revealing the specific function and activity of LTPs in the biosynthesis and evolution of the plant cuticle.

## 1. The Plant Cuticle

Plants colonized land some 500 million years ago [1]. The evolution of molecular barriers formed from lipid-based polyesters was essential for the long-term success of land plants. These barriers protect the plant and also control the fluxes of gases, water, and solutes. The cuticle forms a waterproof layer on the epidermis that is important for protecting plants from biotic and abiotic stresses, such as herbivore attacks, dehydration and radiation [2]. Further, the cuticle is also involved in controlling the morphology of plants [3]. The cuticle is formed from cutin and waxes [2]. Cutin is a lipid polyester, formed mainly from glycerol and long chain (C_16_ and C_18_) hydroxy fatty acids, that in the cuticle are interspersed and covered with cuticular waxes [2,4]. Cuticular wax is a complex mixture of straight-chain C_20_ to C_60_ hydrocarbons, and may include secondary metabolites such as triterpenoids, phenylpropanoids, and flavonoids [5]. 

Synthesis of lipid polymers and cuticular waxes require de novo synthesis of precursors followed by transfer of the precursors through the plasma membrane to the apoplastic compartment [6]. Once exported, the hydrophobic polymer precursors and wax compounds are delivered to the polymerization sites outside the cell wall. This last step is probably the least understood in cuticle biosynthesis. It seems to require that the largely hydrophobic cuticle monomers traffic through the hydrophilic polysaccharide wall to reach the site of cuticle assembly. One family of proteins hypothesized to be involved in this trafficking is the non-specific lipid transfer proteins (LTPs) [4,7]. 

## 2. Lipid Transfer Proteins Could Have a Key Role in Cuticle Biosynthesis

**Figure 1:**
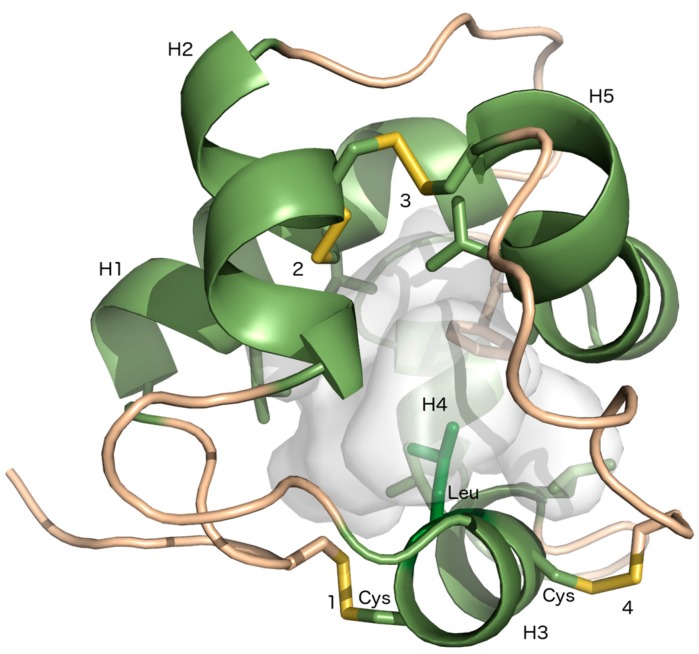
Three-dimensional (3D) model of *Marchantia polymorpha* lipid transfer protein (LTP)g3. The five α-helices of MpLTPg3 delineate the lipid-binding cavity (gray), which is surrounded by hydrophobic amino acids (shown as sticks) [14]. The four disulfide bonds are formed similarly as in LTP2s and the leucine (green sticks) in the CXC motif of H3 points towards the lipid-binding cavity. The figure was created using PyMOL (The PyMOL Molecular Graphics System, Version 1.6 Schrödinger, LLC).

LTPs are soluble, cysteine-rich, and small proteins with a molecular size usually below 10 kDa [8]. They are translated with an N-terminal signal peptide that localizes the protein to the apoplastic space. An LTP protein possesses four or five α-helices, which are stabilized by four conserved disulfide bridges, formed by an eight-Cys motif (8 CM) with the general form C-X*_n_*-C-X*_n_*-CC-X*_n_*-CXC-X*_n_*-C-X*_n_*-C, where X specifies any amino acid and *_n_* is an unspecified number of amino acids. The disulfide bridges promote the folding of the helices around a central hydrophobic cleft (Figure 1), which is suitable for binding of hydrophobic ligands [9,10]. LTPs are compact structures that, to a high degree, are insensitive to heat and denaturing agents [11,12,13]. LTPs are abundant in all investigated land plants, but have not been detected in any other organisms [14]. They are encoded by large gene families in many flowering plants, while in bryophytes and ferns the gene families are significantly smaller [14,15,16,17]. LTPs are classified to one of five major types (LTP1, LTP2, LTPc, LTPd and LTPg) or four minor types (LTPe, LTPf, LTPh, LTPj and LTPk) [14]. The classification is based on the spacing between the Cys residues in the 8CM, the polypeptide sequence identity and the position of evolutionary conserved introns. The classification also reflects post-translational modifications, e.g., LTPs with a glycosylphosphatidylinositol (GPI)-anchor belong to LTPg. LTPd and LTPg were possibly the first LTP types that evolved in land plants, whereas LTP1 and LTP2, the most abundant LTP types in flowering plants, are not found in liverworts, mosses, or other non-seed plants [7,14]*.*

There are a number of features that support the LTPs as stron candidates for delivering hydrophobic cuticle compounds to the apoplastic space: LTPs are synthesized with a signaling peptide and are secreted into the apoplast [7]. They are also abundantly expressed in the epidermis [18,19], small enough to traverse the pores of the cell walls, and their hydrophobic pocket is capable of binding long-chain fatty acids [20]. There is also some experimental evidence supporting a role for the LTPs in cuticular biosynthesis; when gene expression data from rice and Arabidopsis was investigated for co-expression patterns, the LTPgs could be arranged in three co-expressed clusters [21]. For the first cluster (I), expression was observed in aerial parts of the plant. The second cluster (II), was the only one with expression in roots, while expression of the third cluster (III) was restricted to reproductive tissues. Gene ontology analyses of genes coexpressed with the three Arabidopsis LTPg-clusters showed for cluster I an enrichment of genes involved with cuticular wax accumulation, for cluster II an enrichment of genes involved with suberin synthesis or deposition, and for cluster III an enrichment for genes acting in sporopollenin accumulation [21]. These coexpression patterns suggest that the LTPgs in the three clusters are involved in the assembly of the cuticle, suberin and sporopollenin, respectively. 

In Arabidopsis At*LTPg1* and At*LTPg2*, which both encode GPI-anchored LTPs, are highly expressed in the epidermis of inflorescence stems and silique walls [22,23], suggesting a role in cuticle development. Knock-down of At*LTPg1* resulted in reduced wax load on stem surfaces [22]. In At*ltpg1* and At*ltpg2* knock-out mutants, there was a 4–20% reduction in stems and siliques of the C29 alkane (nonacosane) component of cuticular wax, while an At*ltpg1* At*ltpg2* double mutant showed even stronger reductions [23,24]. There was also less total wax load in the stems and siliques of the double mutant and in the siliques of the *ltpg2* single mutant [23,24]. Overexpression of the *Brassica rapa BrLTPd1* gene in *Brassica napus* caused reduced wax deposition on leaves and morphological changes of leaves and flowers [25]. It was speculated that overexpression of *BrLTPd1* leads to disordered secretion of wax, which was then lost from the surface, or to inhibition of other *LTPs* [25].

It is still unclear how LTPs aid in the extracellular transport of building blocks for lipid polymer synthesis. In previously suggested models, ATP-binding cassette (ABC) transporters move the cuticle polymer compounds through the plasma membrane [26,27,28,29]. On the extracellular side of the plasma membrane, lipids are possibly transferred from the ABC transporters to LTPs [7]. Hypothetically, LTPs could stimulate the diffusion or transport of lipid polymer and wax components to the sites of cuticle accumulation on the extracellular side of the plasma membrane [30], which for instance could be the surfaces of leaves, stems or pollen. It is possible that the ABC transporters deliver the polymer building blocks to LTPgs, which are attached to the apoplastic side of the plasma membrane through their GPI-anchor. The cargo may then be transferred from an LTPg to an LTP of another type that may diffuse freely in the cell wall. 

An alternative hypothesis for the role of LTPs in cuticle assembly comes from investigations of AtLTP2 [31]. We previously renamed AtLTP2 to AtLTP1.4 to emphasize that it is of type LTP1 [7], and will use AtLTP1.4 onwards in this review. At*LTP1.4* is expressed only in epidermal cells of aerial organs, and an At*ltp1.4*-mutant has an increased cuticle permeability. In comparison to wild type, the At*ltp1.4*-mutant shows only minor differences in cuticular wax composition. However, in this mutant there is a 30% increase in 18:2 dicarboxylic acid, which is a major cutin component. The At*ltp1.4-*mutant also shows structural defects at the cell wall–cuticle interphase [31]. It was therefore proposed that AtLTP1.4 could play a major structural role by maintaining the integrity of the adhesion between the mainly hydrophobic cuticle and the underlying hydrophilic cell wall [31]. In another study, seeds from several Arabidopsis LTPg loss-of-function mutant lines showed increased permeability to tetrazolium salt, which suggests a malfunctioning seed coat. Morphological seed coat alterations were also shown for several of these *LTPg* mutant lines, as well as seed coat lipid polyesters with increased levels of unsubstituted fatty acids and decreased levels of *ω*-hydroxy fatty acids [32].

**Figure 2:**
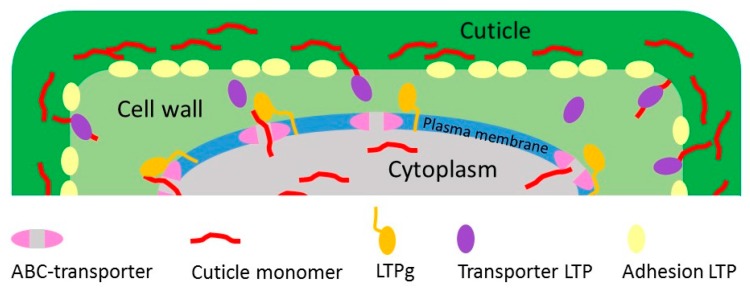
A schematic model for the functions of different LTPs in cuticle assembly. The figure shows three different roles for LTPs in cuticle development. LTPg (orange) is attached to the plasma membrane through its’ GPI-anchor and dock the cuticle monomer when it is leaving the ABC transporter. The Transporter LTP (purple) is then transferring the cuticle monomer from the LTPg through the cell wall to the site of cuticle polymerization [6,20]. The Adhesion LTP (yellow) has a structural role adhering the hydrophobic cuticle to the hydrophilic cell wall, as previously suggested in [28].

Hence, there are rather different roles suggested for LTPs in cuticle assembly and biosynthesis. LTPs may facilitate the transport or diffusion of the hydrophobic cuticle monomers and waxes in the hydrophilic cell wall, such LTPs could be functionally classified as Transporter LTPs (Figure 2). LTPs may also stabilize the adhesion between the cuticle and the cell wall. We classify these LTPs as Adhesion LTPs (Figure 2). These different hypotheses on the function of LTPs do not necessarily contradict each other. In the light of the variety of different LTP-types and the large number of members in the LTP family, it is likely that distinct members of the LTP family could participate as Transfer LTPs or Adhesion LTPs. It is also possible that both functions in transfer and adhesion could be fulfilled by singular LTPs. Further studies will hopefully reveal whether particular LTPs are involved in separate and specific processes during cuticle biosynthesis. 

## 3. The Cuticle in Mosses and Liverworts

Already in the first half of the 20th century, the Finnish botanist Hans Buch described cuticles and waxy surfaces of mosses and liverworts [33]. In 1975, Schönherr and Ziegler [34] used scanning and transmission electron microscopy (SEM and TEM) and histochemistry to show beyond doubt the cuticle at the air pores of the thalli of several liverworts, such as *Marchantia polymorpha*, *M. paleacea*, and *Plagiochasma elongatum* [34]. Four years later, the leaf surfaces of 43 species of mosses were examined with SEM. Twelve species showed well developed superficial wax comparable to the cuticular wax of flowering plants [35]. This study was followed by one of the first reports on the chemical nature of the surface waxes of mosses, as the gametophytes of *Andreae rupestris*, *Pogonatum aloides*, and *Pogonatum urngerum* were shown to contain surface waxes in amounts of 0.05–0.1% of dry weight [36]. The main components of the surface waxes in these mosses were esters, free fatty acids, alcohols, aldehydes, and alkanes. The carbon chain lengths for these compounds were mainly C_20_–C_28_ in length for free fatty acids, C_20_–C_24_ for fatty acid esters, and C_24_–C_28_ for free alcohols and ester alcohols. 

Also, the sporophyte, at least in some mosses, is covered by a cuticle, as shown by surface analysis of the sporophytes of *Buxbamia viridis* [37]. The calyptra is the small cap of maternal gametophyte tissue that covers the top of the sporophyte during development. This structure is covered with a multi-layered cuticle, similar in structure to the cuticle in flowering plants, as shown by SEM and TEM of the calyptra from the moss *Funaria hygrometrica* [38]. Hence, it appears that most aerial surfaces of bryophytes such as the sporophytes and the thalli of liverworts and the gametophores of many leafy mosses are protected by a cuticle with a similar composition and structure as the cuticle in vascular plants.

In vascular plants, the cuticle is a dynamic structure that can be structurally and chemically modified according to environmental and development requirements [39]. There are not many published experiments that address the physiological function and regulation of the cuticle in mosses. However, removal of the calyptra cuticle had a negative impact on development and reproduction. Without the calyptra cuticle, dehydration disrupts sporophyte maturation resulting in decreased spore production [40]. Although it is clear that the cuticle forms a protective barrier on outer surfaces also in liverworts and mosses, we lack substantial information about any dynamic modifications of the bryophyte cuticle in response to developmental or environmental cues. 

Lipid profiling experiments of gametophores of the moss model organism *Physcomitrella patens* have revealed the presence of both cutin and cuticular waxes [13,41]. We have previously reported that unsubstituted fatty acids, fatty alcohols and ω-hydroxylated fatty acids are represented among the moss cutin monomers [13]. Generally, these compounds had chain-lengths of C_16_ or C_18_. The unsubstituted fatty acids were the largest class, with 70% of the total monomer content, and among the hydroxylated fatty acids, only C_16_ were found. There were no dicarboxylated fatty acids found in the moss samples. This class constitutes about 50% of the total cutin monomer content in Arabidopsis, but is significantly lower in other plants [42,43,44]. 

Furthermore, Buda and coworkers [41] identified large amounts of phenolic monomers, such as *m*- and *p*-coumaric acid, and caffeic acid, among the moss cutin monomers. Phenolic monomers are not usually found in the cutin from vascular plants, but are important components of suberin. The presence of phenolic monomers in the moss gametophyte shows that there are clear chemical differences between the cutin of vascular plants and bryophytes. Furthermore, as already pointed out by others, this could indicate that suberin and cutin in vascular plants share a common evolutionary origin [45,46]. The bryophyte cutin could represent a primitive cutin, similar in structure and chemical composition to the lipid barrier polymer of the earliest land plants.

## 4. Mosses and Liverworts as Model Systems for Cuticle Function and Assembly

In recent years, the genomes of several bryophytes, such as the mosses *P. patens* and *Sphagnum fallax*, as well as the liverwort *M. polymorpha* have been sequenced [47,48,49]. Today, *M. polymorpha* and *P. patens* are established land plant model species with an array of available tools for genetic, developmental and molecular analyses [50,51,52]. Because the dominant phase of the bryophyte life cycle is the haploid gametophyte, the effects of transgenes can be studied already a few weeks after transformation. There are established protocols for obtaining gene knock-outs or knock-downs via RNAi, Transcription activator-like effector nucleases (TALENs), CRISPR/Cas9, homologous recombination or artificial miRNAs [53,54,55,56,57,58,59]. There are also many reporter genes and promoters available for gain-of-function, gene expression and protein localization studies [60,61,62,63]. *M. polymorpha* is dioecious, which facilitates the combination of genotypes via crosses. Finally, many liverworts, such as *M. polymorpha*, can reproduce asexually via disc-like propagules, called gemmae, produced in cups on the dorsal side of the thallus. A single plant will start forming gemmae already after a few weeks of growth, and can produce thousands of identical offspring in a short time frame. Hence, mosses and liverworts are already attractive models for advanced molecular studies of fundamental plant biology such as phytohormone signaling, cell growth, stress tolerance, circadian clock responses, and photoreception, just to give a few examples [64,65,66,67,68]. 

**Figure 3:**
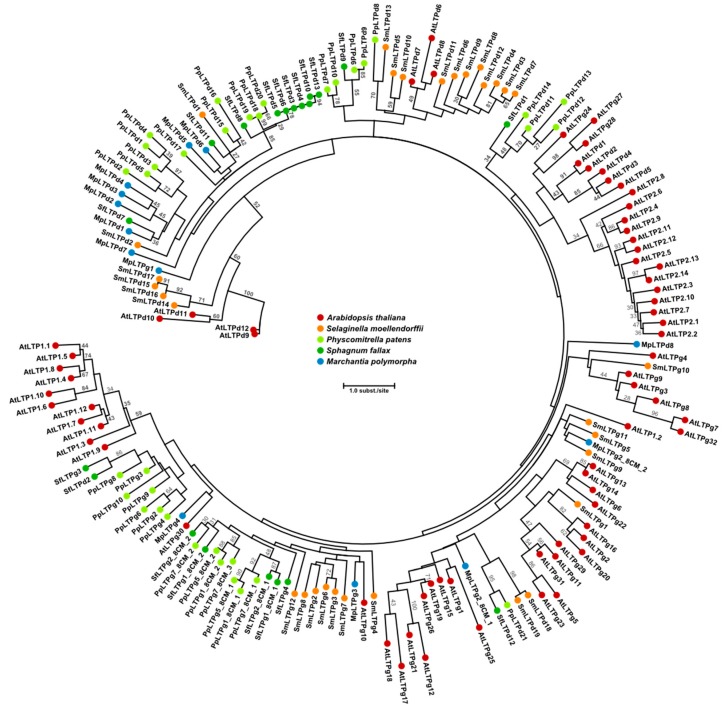
Maximum likelihood phylogram of LTP1, LTP2, LTPd and LTPg proteins from the bryophytes *M. polymorpha*, *S. fallax*, *P. patens*, the lycophyte *S. moellendorffii*, and the spermatophyte *A. thaliana*. Note that LTP1 and LTP2 are not found in bryophytes or lycophytes. *S. fallax* LTPs were predicted using KAPPA [73] on genome annotations available at Phytozome v. 12.1 (https://phytozome.jgi.doe.gov/). Other LTPs are from [14]. Amino acid sequences of the 8CM motif were aligned with MUSCLE [74]; the phylogenetic tree was constructed using PhyML 3.0 [75,76] with 100 bootstrap replicates. Bootstrap values over 25 are shown in the figure.

Mosses and liverworts are emerging as models for studies on the assembly, function and evolution of the plant cuticle [14,41,46]. For instance, it will be of interest to apply these bryophyte model systems to investigate the details of the function of LTPs in cuticle biosynthesis and development. Genome analyses have shown that LTPs were present in the ancestors of extant land plants [14]. In *M. polymorpha* we previously identified 14 LTP genes. Eight of these belong to the LTPd subgroup (12 in Arabidopsis [7]), while four genes correspond to LTPg (29–34 in Arabidopsis [7]; Figure 3)*.* When classified according to the criteria mentioned above, the two remaining LTP sequences in *M. polymorpha* did not fit into any of the major or minor LTP types. A genome-wide search revealed that a total of 40 LTPs are found in *P. patens* [14]. The LTP content in *P. patens* and also *S. phallax* qualitatively resembles that of the liverwort *M. polymorpha*; LTPd and LTPg are the dominant types and constitute the main part of the LTPs (Figure 3) [7,14]. Hence, the number of LTP genes is much lower in bryophytes compared to vascular plants, suggesting less redundancy, in a similar manner as shown for many other gene families in *M. polymorpha* [49]. The low gene copy number and putatively low redundancy, together with the fast generation of loss- and gain-of-function mutants, suggests it might be easier to obtain conclusive results on the function of specific subgroups, i.e., LTPd and LTPg, in *M. polymorpha* than in other species. 

The only bryophyte LTPs that have been studied in some detail are PpLTPg2 and PpLTPg8 from *P. patens*. These proteins show many similarities to LTPs from higher plants in terms of lipid binding, gene regulation, thermal stability and three-dimensional (3D) structure. According to 3D-modelling, these proteins have the LTP2-type of 8CM, with disulfide parings between Cys residues 1-5, 2-3, 4-7 and 6-8 [13]. In competition assays, PpLTPg2 and PpLTPg8 showed a preference for unsaturated C18 fatty acids, while seemingly saturated or longer fatty acids were not as easily fitted in the hydrophobic binding cavity. Similar results have been obtained for several LTPs from vascular plants [69,70]. When the expression patterns of all eight PpLTPg genes were analyzed, it was found that drought caused a significant upregulation of the majority of the genes. Drought also upregulates the expression of LTPs in Arabidopsis and rice [71,72]. Many of the *cis*-elements identified in moss promoters were also previously identified in Arabidopsis [21], suggesting that regulatory circuits controlling LTP expression are conserved between mosses and flowering plants. To summarize, it is clear that LTPs from mosses share many features with LTPs from vascular plants. It therefore seems likely that bryophyte and seed plant LTPs are involved in similar or related physiological processes.

In this review we have focused on the use of bryophyte model systems to identify the role of LTPs in cuticle assembly. We have argued that LTPs are involved in cuticle assembly in plants, that bryophyte LTPs have similar properties and likely related functions to LTPs from seed plants, and that bryophytes could be useful model organisms for research on the plant cuticle. The challenge now is to design experiments that will confirm or reject a role for LTPs in cuticle assembly.
